# Extensive reforms to improve funding efficiency: an interview with President Xiankang Dou of National Natural Science Foundation of China

**DOI:** 10.1093/nsr/nwae123

**Published:** 2024-05-21

**Authors:** Weijie Zhao, He Zhu

**Affiliations:** NSR in Beijing, China; NSR in Beijing, China

## Abstract

Dr. Xiankang Dou (窦贤康) was appointed the President of the National Natural Science Foundation of China (NSFC) in April 2023. As a distinguished researcher specializing in studying the middle and upper atmosphere of earth, Dr. Dou had served as the President of Wuhan University during 2016 to 2022 and was elected an academician of the Chinese Academy of Sciences (CAS) in 2017.

At his new post in NSFC, Dr. Dou's first priorities include curtailing personal favors in the review process (often known as ‘say hello’ with an intent to lobby), reforming the scoring and renewing mechanisms for the National Science Fund for Distinguished Young Scholars (DYS), opening the channel for DYS applications to researchers from Hong Kong and Macau, extending the age limit of female scientists for applying for DYS, and initiating funding programs for outstanding doctoral and undergraduate students. Recently, *National Science Review* (NSR) had an interview with Dr. Dou. He believes only through reform and experimentation can NSFC make steady progress in its efforts to support research of basic science and applied basic science in China and to cultivate promising scholars.

## Teaser

NSFC President Xiankang Dou believes only through reform and experimentation can NSFC make steady progress in supporting research of basic science and applied basic science in China and cultivating promising scholars.

## THE MISSION AND FUNDING STRUCTURE OF NSFC

**NSR: In 2023, China reorganized its Ministry of Science and Technology and formed the Central Commission of Science and Technology. How do these changes affect NSFC?**

**Dou:** The strategic positioning of NSFC was specified in 2014 by the *Management Blueprints of Extensive Reforms in Financial Planning for Science and Technology*. The missions of NSFC include funding basic research and scientific exploration, cultivating research talents and teams and promoting breakthrough innovations. After recent institutional reforms of the State, two branches in the Ministry of Science and Technology came under the management of NSFC. They are the High-Tech Research and Development Center and the Administrative Center for China's Agenda 21.

These changes represent the State's systematic allocation of basic research and applied basic research based on the principles of research development and duties of NSFC in this new era. With consolidated resources, NSFC now operates more effectively as the primary source of support for basic research and applied basic research and plays a greater role in elevating the quality and innovation in research activities.

**NSR: What are the primary categories of funding at NSFC? How do they form a comprehensive funding structure?**

**Dou:** NSFC currently operates 18 funding programs, sorted into three categories.

Exploratory programs in the first category include General, Key and Major Programs. This category aims to encourage scientists to explore and try new ideas in their fields of interest. Major breakthroughs in science often originate from unexpected ideas. An important duty of NSFC is to provide broad support of general programs with ample durations and stability. A special emphasis is to maintain a wide coverage of all scientific disciplines.

**Figure 1:**
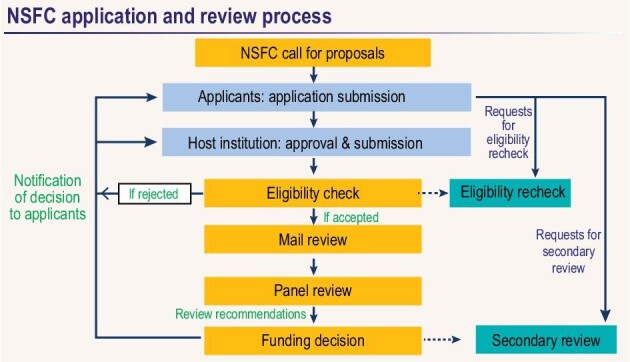
NSFC application and review process.

Talent programs in the second category include Distinguished and Excellent Young Scholar (DYS and EYS) programs as well as Creative Research Groups and Basic Research Center programs. This year, we have also added funding options for doctoral candidates and undergraduate students. We hope this series of talent programs together shall form a continuous and systematic funding mechanism, through which scientists build their careers from the undergraduate stage all the way up.

Environmental programs in the third category provide support for developing scientific instruments and building research platforms. NSFC does not invest in research and development of general instruments. Instead, NSFC only supports original designs aimed at solving specific scientific problems.

## PREVENT FAVOR-CALLING, SAFEGUARD FAIRNESS

**NSR: In terms of safeguarding fairness in the review process and reducing the instances of ‘saying hello’, what are the new policies at NSFC?**

**Dou:** NSFC launched preventive measures against favor-calling in the grant review process in 2023. We put the essence of them into a few words: positive guidance, unequivocal definition, strict prevention, and serious consequences. Positive guidance refers to lectures and other activities of public outreach. We hope to let researchers understand the severity of corruption in science and voluntarily refuse to call in personal favors.

In terms of ‘unequivocal definition and strict prevention’, we took multiple actions. For example, we posted a List of Forbidden Actions of Favor-calling during NSFC Reviews where we clearly define the actions that are not allowed. In the process of working with reviewers to implement these policies, we curtailed the effects of favor-calling.

In terms of ‘serious consequences’, we enacted harsh punishments whenever a breach of these rules was detected and verified. Such punishments can be as severe as permanent loss of eligibility as a reviewer or an applicant in any NSFC program.

Excellent results have been delivered after these policies were enacted. According to an independent survey, the incidence of favor-calling was greatly reduced. Our next step in the process of combating favor-calling is to summarize our success and establish a systematic mechanism that monitors the entire review process. We aim to strengthen the aspect of positive guidance and maintain the public awareness of this issue. We are building a longstanding culture where favor-callings are mostly ineffective.

We posted a List of Forbidden Actions of Favor-calling during NSFC Reviews where we clearly define the actions that are not allowed.

—Xiankang Dou

## REFORMS IN TALENT PROGRAMS

**NSR: After taking the helm at NSFC, you have made changes in talent programs. Can you tell us more about it?**

**Dou:** I joined NSFC with one clear objective: allocating limited resources of the State to support the most productive scientists on the front line. We proposed a series of reforms such as continued support for DYS and trial funding programs for doctoral candidates and undergraduate students. In addition, we increased the presence of young reviewers for NSFC-funded projects. In NSFC program reviews for the year 2023, at least a third of reviewers were younger than 45.

**NSR: The reforms made in the DYS program generated some discussions. Please tell us more about it.**

**Dou:** We made major reforms in the DYS program, and their implementation will start in 2024. By highlighting its ‘project attributes’, NSFC will provide continuous support to the principle investigators (PIs) of the DYS program that perform well. The emphasis now is on project outcome classification and continued support of top-tier recipients. Specifically, we now conduct project evaluations at the end of the funding period and assign final scores of *Excellent, Good* or *Poor*. Each score will be used by a recipient's research institution as a reference. Projects scoring *Excellent* (top 20% of all) will get doubled funding up to 8 million yuan in the next 5 years. At the end of the second funding period, the top 50% of recipients will be awarded 16 million yuan for the third five-year period to help them grow into leaders of their fields.

These policies aim to accomplish two goals. First, we want to provide long-term support to the researchers that are proven to be excellent. Second, we want to reduce, to some extent, the influence of the initial award (becoming a title or a lifelong honor) so recipients will maintain their motivation. After receiving the initial award, the recipients should realize it is only a new beginning and more research and innovation are expected from them.

Another change is that we plan to open the DYS program to Hong Kong and Macau in the same fashion as the EYS and Young Scientist program. The applicants and their corresponding institutions are to be reviewed and supported according to the same standards as those in mainland of China. All applicants will compete fairly and openly on the same platform. This will further strengthen the support of research talents of basic sciences in Hong Kong and Macau.

**NSR: The undergraduate funding program received wide attention when it started in 2023. How is it implemented and what are the goals?**

**Dou:** In this trial program, we hope to select excellent undergraduate students that are capable of more work apart from their daily study and plan to have careers in research. We want to help them get used to thinking independently. We will reach our goal if they become more motivated to conduct independent research when the program concludes. After the program started, we discovered that there are indeed a group of excellent undergraduate students that deserve funding. To evaluate and help these students, we also organized a team of experts from the research front line, including mostly recent DYS grantees. These experts participate in this program from selection to completion to ensure continuous oversight. In addition to financial support, we also support their professional growth such as attending academic conferences.

We picked 129 students in eight universities to roll out the undergraduate program. We are also expanding our support to doctoral students and in the first round we picked 23 universities. These early-stage support programs will possibly foster some first-class scientists for China.

**NSR: In 2023, NSFC also increased support of female scientists. What are the new policies?**

**Dou:** Starting from 2024, the age limit of female applicants for DYS will be extended to 48. In the review process, favorable policies are also adopted for female applicants with comparable credentials. Women take on more duty at home and in society, so they face more difficulty at work. This reality makes it correct and necessary to implement policies to compensate for the disadvantages. In recent years, more and more excellent female scientists have emerged around the world and we hope female scientists in China will receive more support and achieve more success.

**NSR: What is your reaction to criticisms generated by the reforms at NSFC?**

**Dou:** It is inevitable to have disagreements in the process of implementing reforms. We should maintain a positive and open mind. Some criticism and suggestions may provide us with important insights to improve our work and optimize our reform plans. They may also encourage us to invest more effort to fine-tune every step of our work. The reforms we are making came from extensive surveys and analysis of our funding records. China's economic reforms and opening up, which have made great achievements, have taught us one most valuable lesson: trials and explorations before consensus may lead to optimal solutions. I believe the reforms we are making today, at least some of them, will be proven effective and right in the future.

By highlighting its ‘project attributes’, NSFC will provide continuous support to the PIs of the DYS program that perform well.

—Xiankang Dou

## INCREASE INTERNATIONAL FUNDING AND EXCHANGE

**NSR: Research of basic sciences relies on international collaborations. What is NSFC doing to encourage more collaborations?**

**Dou:** Research of basic sciences in China has benefited from international collaborations in the past decades. As we improve, we start to play more important roles internationally. First, we strive to be more open to international collaborations and build a platform conducive to basic research collaboration. Second, we continue to conduct dialogues on funding policies with international partners and we actively participate in global governance of science and technology.

NSFC just established the Department of International Programs in September of 2023. It serves two functions. First, this department supports research projects consisting of international collaborations. Second, it invites scientists from overseas to conduct extensive research in China. We recently started communications with the Ministry of Education and plan to jointly fund doctoral candidates from other countries to conduct research and get a degree in China. In the past 100 years or more, Chinese students travelled to the West to study. Now as China develops in science with better facilities, we also welcome and support excellent foreign students to come to China to study and conduct research. In conclusion, NSFC is committed to support our scientists and those overseas to collaborate on basic sciences.

## DEPARTMENT OF INTERDISCIPLINARY SCIENCES SEES EARLY SUCCESS

**NSR: NSFC established the Department of Interdisciplinary Sciences in 2020. How is it operating so far?**

**Dou:** Interdisciplinary research is becoming the mainstream so starting this department is a wise decision. Now we provide better support to scientists in interdisciplinary fields. For example, a DYS applicant this year is working on the connection between the brain and human behavior. His chance in the Department of Life Sciences may not be good because his research focuses on brain regions, not reaching the cellular or molecular level. However, his work was recognized and funded by the Department of Interdisciplinary Sciences to combine the research in medical sciences and social sciences.

In the Department of Interdisciplinary Sciences, we are trying new policies and review guidelines that are tailored to fit the unique characteristics of interdisciplinary research. For example, interdisciplinary projects involve more topics, requiring a wide range of experts to review. A crucial problem in this process is to ensure reviewers from related fields evaluate an applicant's professional expertise and general reviewers evaluate his or her comprehensive ability in science. To address this issue, we implemented an ‘interactive defense’ that allocates less time to project presentation and more time to question-and-answer. In the course of thorough communication, reviewers from related fields posed questions of professional details while reviewers from the broader disciplines estimated an applicant's scientific background and general research training. This format helps to present an applicant's vision from multiple aspects so that a comprehensive opinion can be reached.

The Department of Interdisciplinary Sciences will continue to explore new methods in future work to build a review and funding model that fits the development of interdisciplinary sciences in China.

## SERVE THE PUBLIC WITH RESEARCH DISCOVERIES

**NSR: In terms of setting up Joint Funds and building partnerships to support basic research with diversified sources, what is NSFC doing?**

**Dou:** The Joint Fund is an excellent program designed by NSFC. So far, 29 local governments have participated in the Joint Fund for Regional Innovation and Development and 12 state-owned enterprises have participated in Joint Fund for Enterprise Innovation and Development. Nine industrial sectors have set up 10 Joint Funds with NSFC. During the implementation of a joint fund, the funding partner plays the role of ‘questioner’ and ‘examiner’. NSFC plays the role of the ‘answerer’, taking full advantage of the scope and depths of our expertise so that we find the most suitable, most innovative research team to address the scientific problems raised by the ‘questioner’.

The Joint Fund has seen great progress in past years and we are planning continuous expansion to a proper scale. We will keep making improvements to the program guidelines and allocate more responsibilities to joint partners during management and final evaluation to further improve funding efficiency.

**NSR: In terms of commercialization, what are NSFC planning to do?**

**Dou:** NSFC has now completed the NSFC Service Platform for Research Commercialization (Gen 1). This platform serves as a bridge to connect researchers, enterprises and the public. It sorts and indexes excellent outcomes of NSFC-funded research into a library, and on the other hand it collects and posts commercial requests from enterprises. In this way it helps to align ‘supply’ and ‘demand’ effectively. The commercialization from NSFC focuses on our Instrumentation programs, Key programs, Major programs and Joint Funds. We initiate collaborations with regional governments through in-person forums. By actively publicizing our funded research, we aim to create commercialization and help the economic growth of local regions.

## Author Biography

Weijie Zhao (赵维杰) and He Zhu (朱禾) are science and news editors at the editorial office of NSR in Beijing, China

